# Barriers to Accessing and Providing Primary Care Services for LGBTIQA+ Individuals Experiencing Intimate Partner Violence: A Scoping Review

**DOI:** 10.1177/15248380251361063

**Published:** 2025-08-30

**Authors:** Jack Farrugia, Danny Della Vedova, Bronwyn Milkins, Jacqueline Hendriks, Sharyn Burns, Roanna Lobo

**Affiliations:** 1 Curtin University, Perth, WA, Australia

**Keywords:** LGBTIQA+, intimate partner violence, primary care service, scoping review, service access

## Abstract

LGBTIQA+ individuals experience intimate partner violence (IPV) more frequently than non-LGBTIQA+ individuals but seek help at lower rates and frequently receive inadequate care from primary care providers. This scoping review identified barriers to accessing and providing primary care services for LGBTIQA+ people experiencing IPV. The review was conducted in accordance with the Joanna Briggs Institute methodology for scoping reviews. Three databases (ProQuest, MEDLINE, and CINAHL) were searched for peer-reviewed literature published in the Organization for Economic Co-operation and Development in the last 20 years before October 2024. Gray literature was identified through a Google and Google Scholar search. Reference lists from relevant reviews were manually searched to identify records that met the inclusion criteria. A total of 5,439 records were identified. After de-duplication and applying inclusion criteria, 19 publications were retained for analysis. Findings indicate that LGBTIQA+ individuals experiencing IPV face barriers to accessing primary care, including difficulty recognizing IPV, fear of discrimination, and systemic inequities such as inadequate provider training and non-inclusive services. Primary care providers report heteronormative assumptions in services, lack of LGBTIQA+ specific training, and institutional resistance to inclusive care. These findings highlight the need for targeted interventions that both encourage LGBTIQA+ individuals to seek IPV-related primary care support and equip providers with the training and resources necessary to deliver culturally competent care, while also addressing the cisgender and heteronormative assumptions that perpetuate systemic inequities in primary care service provision.

## Introduction

Intimate partner violence (IPV) is a public health issue receiving growing attention due to its significant emotional, social, and economic impact on individuals experiencing IPV, their families, and the broader community (Peterson et al., 2018; Stubbs & Szoeke, 2022). Additionally, IPV is a leading contributor to morbidity and mortality, with severe physical and psychological consequences, including homicide and suicide (Kafka et al., 2022). IPV refers to patterns of coercive, abusive, or controlling behaviors that occur within the context of an intimate relationship and lead to sexual, physical, psychological, or emotional harm (Campbell, 2002). IPV is also often referred to as spousal abuse or domestic violence (Campbell, 2002). An intimate partner may be a current or previous partner from a formal or informal relationship, including dating (face-to-face or online), living with or de facto, “hooking up” with, or married to (Campbell, 2002). Since the 1970s, feminist grassroots movements have influenced government action to reform policy and laws to address violence against women (Kevin, 2020).

The feminist basis for the response to domestic violence has contributed to a cisgendered and heteronormative narrative that domestic violence occurs in heterosexual relationships only, in which violence is perpetrated by men against women. This theoretical background has had negative repercussions for the awareness, identification, and response to IPV in lesbian, gay, bisexual, transgender, intersex, queer, and asexual (LGBTIQA+) individuals with a lack of suitable crisis support options or refuges (Calton et al., 2016; Furman et al., 2017). This is concerning, as reports suggest LGBTIQA+ individuals may experience IPV as much, if not more than, cisgender heterosexual women (Bermea et al., 2018, 2021; Hillman, 2022; Peitzmeier et al., 2020; Valentine et al., 2017).

While LGBTIQA+ individuals can experience forms of IPV that are common across cisgender and heterosexual contexts, they also face unique forms of IPV (Walters & Lippy, 2016). Examples include threatening to “out” a partner to friends or family, using homophobia, transphobia, or biphobia to exert coercive control or abuse over a partner, and withholding access to medication or gender-affirming hormones (Brown & Herman, 2015; Walters & Lippy, 2016).

There is a high prevalence of IPV among LGBTIQA+ individuals globally. A range of studies indicates that IPV affects LGBTIQA+ individuals at levels equal to or higher than those observed in heterosexual populations (Callan et al., 2021; Calvet & Cantera, 2023; Hillman, 2022; Juwono et al., 2024; Liu et al., 2021; Rothman et al., 2011; Yan et al., 2024). Studies have also documented high prevalence rates of IPV among subgroups within LGBTIQA+ populations, with bisexual individuals, lesbian women, transgender individuals, and gay men experiencing IPV at concerning rates (Bermea et al., 2018; Callan et al., 2021; Hillman, 2022; Liu et al., 2021; Peitzmeier et al., 2020). Despite the high prevalence of IPV among LGBTIQA+ populations, help-seeking behavior remains low in many countries, with barriers such as stigma, fear of further victimization, and lack of appropriate services (Callan et al., 2021; Calvet & Cantera, 2023; Hillman, 2022; Juwono et al., 2024; Liu et al., 2021; Rothman et al., 2011; Yan et al., 2024). Private Lives 3, the largest national survey of the health and wellbeing of LGBTIQA+ people in Australia, highlights examples of these prevalence rates (Hill et al., 2020). The study found 41.7% (*n* = 2,846) of participants reported feeling abused by an intimate partner, and of these, 28.0% (*n* = 1,325) had reported the most recent incident to a relevant service (Hill et al., 2020).

LGBTIQA+ individuals often experience discrimination based on their sexual or gender identity within health services, which can affect their willingness to seek support in the future (Mehta, 2017). In addition, they experience unique barriers when seeking help for IPV from primary care services, compared to non-LGBTIQA+ individuals (Donne et al., 2018; Freedberg, 2006). These barriers may include difficulties in recognizing their experiences of IPV, as societal norms often focus on the experiences of non-LGBTIQA+ individuals (Brown & Herman, 2015; Cannon, 2015; Walters & Lippy, 2016).

Studies have also reported on systemic inequities, including heteronormative policies and procedures that do not protect LGBTIQA+ individuals from prejudice and discrimination, and a lack of training for staff on providing tailored support to LGBTIQA+ individuals (Donne et al., 2018; Freedberg, 2006). The attitudes and behaviors of primary care providers toward LGBTIQA+ individuals experiencing IPV can also vary widely, from being inclusive and welcoming to heteronormative and exclusionary, making it challenging for LGBTIQA+ individuals to identify safe options for support (Bermea et al., 2018). These barriers to support seeking and service delivery need to be considered and addressed to improve health outcomes for LGBTIQA+ individuals experiencing IPV.

The focus of existing IPV research has primarily been on heterosexual cisgender women (Callan et al., 2021). While this research has played a foundational role in shaping the contemporary understanding of IPV and has provided the context for the development of current primary care settings, it may not offer relevant findings when considering the needs of LGBTIQA+ individuals experiencing IPV (Callan et al., 2021; Calton et al., 2016). There have been few reviews focusing on the help-seeking behaviors of LGBTIQA+ individuals who have experienced IPV (Calton et al., 2016; Freedberg, 2006; Santoniccolo et al., 2023; Wright et al., 2022). These reviews have not specifically explored the essential role of primary care providers or the specific barriers experienced by LGBTIQA+ individuals seeking IPV-related support. This is a concerning gap in the literature, as primary care providers are often the first point of contact for individuals experiencing IPV and are therefore well placed to offer support. There is a need for a review that focuses on the provision of support services by primary care providers to inform the development and integration of evidence-based approaches, helping to reduce the severe physical, psychological, and social consequences of IPV among LGBTIQA+ populations.

### Research Questions

A scoping review is an appropriate method to determine current trends in IPV research in LGBTIQA+ communities and identify gaps in intervention research that need to be addressed. It provides a systematic and rigorous approach to mapping existing literature, making it a valuable method for thoroughly examining issues that have not yet been comprehensively explored. A preliminary search of MEDLINE, the Cochrane Database of Systematic Reviews, and Joanna Briggs Institute (JBI) Evidence Synthesis was conducted, and no current or registered systematic or scoping reviews on the topic were identified.

The objective of this scoping review was to identify barriers to accessing and providing primary care services for LGBTIQA+ people experiencing IPV. Two research questions guided the review:

1. What barriers do LGBTIQA+ individuals face when accessing primary care services for concerns related to IPV?
2. What barriers do primary care providers face in delivering IPV-related care to LGBTIQA+ individuals?

## Methods

This scoping review was conducted in accordance with the JBI methodology for scoping reviews (Peters et al., 2022). This review was also completed and presented following the Preferred Reporting Items for Systematic Reviews and Meta-Analyses (PRISMA) extension for scoping reviews (Tricco et al., 2018).

## Search Strategy, Data Sources, Collection, and Organization of Data

The review aimed to locate original peer-reviewed empirical studies and gray literature. An initial limited search of MEDLINE (Ovid) and PubMed was conducted to identify relevant articles on the topic. The words used in the titles and abstracts of these articles, along with the index terms used to describe them, were utilized to develop a comprehensive search strategy, in consultation with a university librarian.

Database searches were conducted in October 2024 and included ProQuest, MEDLINE (Ovid), and CINAHL. Although both PubMed and MEDLINE were initially consulted to develop the search strategy, only MEDLINE (Ovid) was included in the formal search based on librarian advice, as it contains the majority of PubMed content and offers enhanced indexing. The final search included terminology focusing on IPV and LGBTIQA+ populations. A sample ProQuest search is included in Supplemental Appendix A. The search strategy, incorporating all identified keywords and index terms, was adapted for each database and information source. To identify gray literature sources, the first 100 records in Google Scholar and Google were searched. Reference lists from relevant reviews were manually searched to identify potential records for inclusion.

All identified records were uploaded to EndNote 20 (Clarivate Analytics, PA, USA) and duplicates were removed. These records were then imported to SUMARI (Joanna Briggs Institute, 2020) for screening. Two authors independently screened the titles and abstracts, and disagreements were resolved through discussion. The full texts of the remaining articles were also screened by these reviewers, and any disagreements were similarly resolved.

## Inclusion and Exclusion Criteria

Studies were included if they were in English and published in an Organization for Economic Co-operation and Development (OECD) member country within the last 20 years (since 2004). Studies were excluded if they did not (a) focus on LGBTIQA+ individuals accessing primary care services in the context of IPV, and (b) specifically report on barriers to receiving care for IPV. Primary care services are the first point of contact for individuals seeking healthcare outside of hospital or specialist settings (Australian Institute of Health and Welfare, 2025; World Health Organization, n.d.). Primary care providers include general practitioners, psychologists, social workers, physiotherapists, occupational therapists, and specialist medical providers such as gynecologists (Australian Institute of Health and Welfare, 2025; World Health Organization, n.d.). Services delivered by primary care providers include diagnosis and treatment, crisis counseling, health promotion and education, and preventive services (Australian Institute of Health and Welfare, 2025; World Health Organization, n.d.). Types of primary care services include general practice, community health centers and walk-in clinics, community pharmacies, community nursing services, oral health and dental services, mental health services drug and alcohol treatment services, sexual and reproductive health services, maternal and child health services, and allied health services (Australian Institute of Health and Welfare, 2025; World Health Organization, n.d.). Studies involving services outside the primary care system, such as law enforcement or judicial services, were only included if primary care services were also included and findings specific to primary care services could be extracted. This was also applied to cases where support services were discussed in general terms or when the type of service was not specifically identified. Similarly, studies exploring IPV and support service interactions among non-LGBTIQA+ individuals were only included if the primary context centered on LGBTIQA+ individuals’ experiences with primary care services for IPV. In these cases, only information relevant to the topics were extracted.

## Mapping, Extraction, and Synthesis

Data on the study characteristics and key findings relevant to the review questions were extracted in an Excel table. Thematic analysis (Braun & Clarke, 2022) was used to identify the barriers to accessing and providing primary care services in the context of IPV and LGBTIQA+ populations. The analysis was inductive to allow the themes to be naturally derived from the data in the included studies (Braun & Clarke, 2022). The researchers familiarized themselves with the included studies and initial codes, and subsequent themes.

## Results

**Figure 1.**
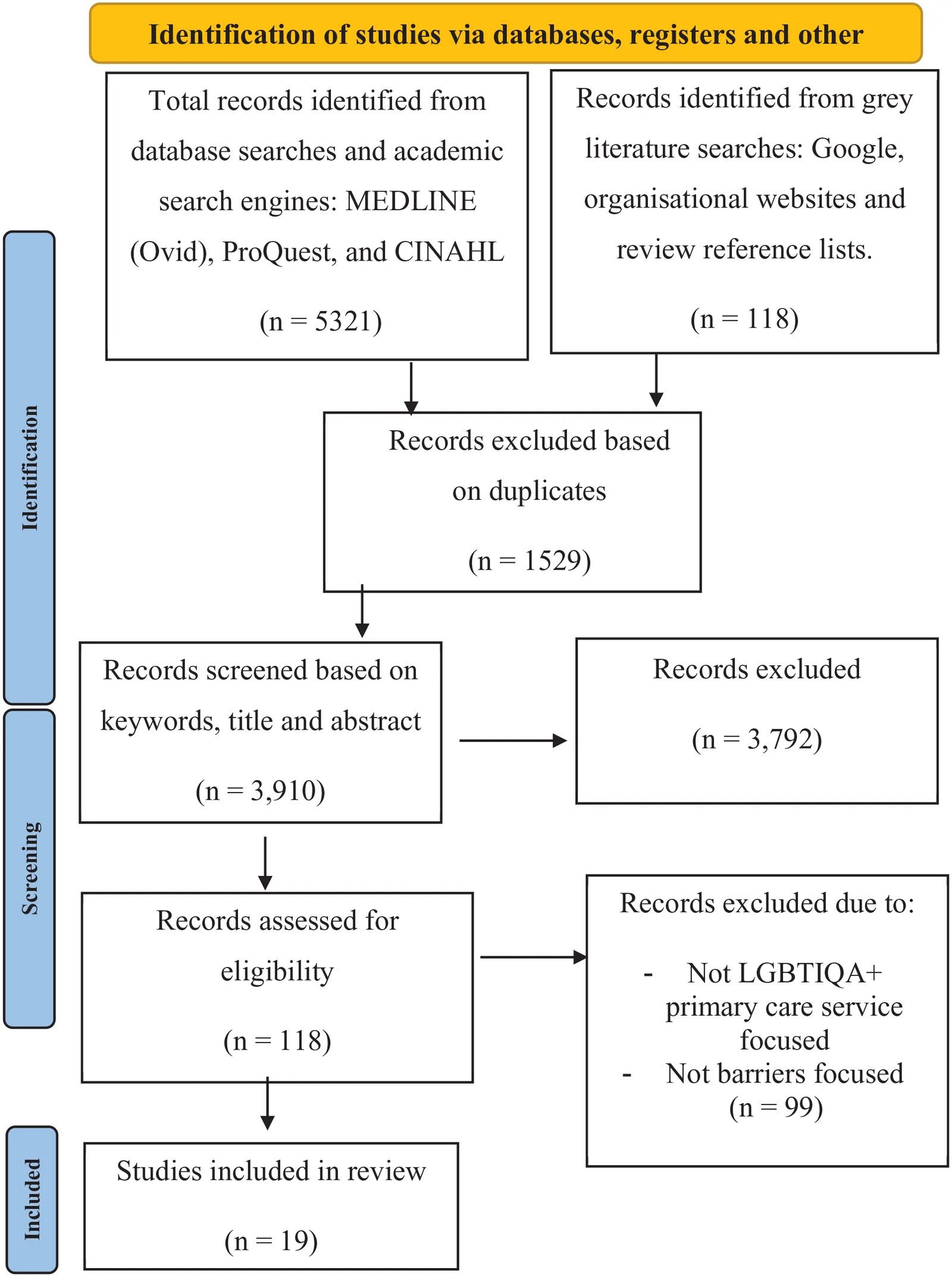
PRISMA flow diagram. *Note*. PRISMA = Preferred Reporting Items for Systematic Reviews and Meta-Analyses.

As presented in Figure 1, the database search resulted in 5,321 records, and 118 records from the gray literature searches, totaling 5,439 records. After removing 1,529 duplicates, the titles and abstracts of the remaining 3,910 records were screened against the inclusion and exclusion criteria. Subsequently, 3,792 records were removed, leaving 118 records for full-text screening. Of these, 99 records did not meet the criteria and were removed. Nineteen records were retained for analysis. These included 13 studies that focused on the barriers LGBTIQA+ individuals experience in accessing primary care support for IPV and 6 studies that examined the barriers primary care providers experience in delivering IPV support to LGBTIQA+ individuals. These studies were analyzed separately. The LGBTIQA+ acronym has been used to encompass all subgroups referenced in the findings; however, we acknowledge that this may be inaccurate, as not all studies included every subgroup. See Tables 1 and 2 for the study characteristics and findings.

**Table 1.** Study Characteristics and Key Findings on Barriers to Primary Care Access for LGBTIQA+ Individuals.

Authors (Year), Country	Design	Sample Characteristics	Primary Care Service	Findings
Das et al. (2022), USA	Quantitative (Retrospective Electronic Health Record [HER] Review)	Transgender patients (*n* *=* 1947)	Primary care services	Anticipated transphobia and discrimination deter help seeking
D’Cruz et al. (2024), NZ	Mixed methods (survey and interviews)	LGBTQIA+ individuals (*n* = 363)	Formal support services are privatized support systems and services that have specialized training for IPV (e.g., counselors, medical professionals, domestic violence organizations, law enforcement)	Formal supports are seen as inaccessible and unsafe; heteronormativity linked to shame in help seeking; fear of discrimination; lack of competency working with LGBTQIA+ individuals; disclosure concerns deter help seeking
Donne et al. (2018), USA	Qualitative (interviews)	Straight gay, cisgender, and transgender men (*n* = 32)	General support services (including mental health counseling, therapy, and support groups) and sexual violence-specific support services	Barriers include: lack of IPV awareness, gender norms; perceived severity; financial costs; scheduling; trust issues; and inadequate intersectional support
Donovan and Barnes (2020), UK	Mixed methods (survey and interviews)	Lesbian, gay, bisexual, and transgender victims/survivors (*n* = 872)	Privatized sources (counselors/therapists)	Fear of provider misunderstanding, blame, or dismissal; concerns about reinforcing negative LGBTQ stereotypes; heteronormativity in services discourages help seeking
Kurdyla (2023), USA	Qualitative (interviews)	Transgender survivors (*n* = 9)	Support services including healthcare practitioners and counselors	Anticipated and experienced transphobia discourages disclosure; self-blame due to internalized transphobia; difficulty recognizing IPV experiences
Martin et al. (2022), USA	Quantitative (survey)	Lesbian, gay, bisexual, and heterosexual victims (*n* *=* 1308)	Formal support services, comprising police, psychological care, a victim services agency, and medical assistance	Help seeking is influenced by service availability and expectations of provider treatment
Morris et al. (2024), USA	Mixed methods (survey and interviews)	LGBTQ people of color individuals (*n* = 263)	Health care services (non-specific)	Lack of IPV screening; concerns about disclosing LGBTQ identity; providers overlook LGBTQ survivors’ needs; trust and identity concordance critical
Ovenden et al. (2019), AUS	Mixed methods (survey)	GBTIQ men (*n* = 895)	Mental health and medical	Men struggle to recognize IPV; Younger men minimize IPV
Palich et al. (2024), UK	Qualitative (interviews)	Cis-gay, bisexual, and men who have sex with men, and transgender people (*n* = 15)	Health care services (non-specific)	Negative experiences with clinical staff post-IPV; confidentiality and empathy issues
Richardson et al. (2015), USA	Mixed methods (survey)	LGBQ individuals (*n* = 2482) bisexual, 88	Formal support, including counseling centers and health services	LGBQ survivors perceive IPV as less serious; fear of blame and self-blame due to drinking; lack of IPV awareness and education
Scheer et al. (2023), USA	Quantitative (survey)	Sexual and gender minority adolescents and young adults assigned female at birth (*n* = 193)	Support services (not specific)	Most common barriers: minimizing IPV; fear of judgment/blame/disbelief; doubts about service effectiveness
The Australian Centre for Social Innovation (2018), AUS	Qualitative (workshops)	LGBTI community members (*n* = not specified)	Mainstream services for IPV or family violence. Counselors and therapists. Doctors and medical health professionals. Trans-specific health and medical professionals. Queer-friendly services and supports	IPV is unrecognized due to LGBTI invisibility in family violence discourse; lack of inclusive services; binary service access criteria exclude LGBTI individuals
Turell and Cornell-Swanson (2005), USA	Quantitative (survey)	LGBT individuals (*n* = 677)	Counseling, police, shelters, DV agency, crisis hotline, medical, legal	Gay men feel embarrassed to disclose; bisexual victims feel disconnected; lesbians downplay IPV; transgender survivors prioritize other issues

**Table 2.** Study Characteristics and Key Findings on Barriers to Delivering Support Among Primary Care Providers.

Authors (Year), Country	Design	Sample Characteristics	Primary Care Service	Findings
Bytheway and Stephens-Lewis (2024), UK	Qualitative (interviews)	Therapists (*n* = 5)	Mental health, private practice, addiction, domestic abuse, and children’s services;	Heteronormativity dominates IPV response; LGBTIQA+ clients internalize shame; false claims of inclusivity; burden of educating providers placed on clients.
Du Mont et al. (2024), CAN	Quantitative (survey)	Healthcare or social/community service providers (including coordinator, educator, executive, frontline, provider, manager, support staff, and volunteers) (*n* = 163)	Healthcare and social/community services	Lack of training, curriculum, and resources for supporting transgender and gender-diverse IPV survivors.
Furman et al. (2017), CAN	Qualitative (interviews)	Professionals (including counselors, shelter managers, program directors) (*n* = 10)	Organizations that provide services for IPV survivors	Mainstream services fail to be inclusive; current training insufficient; systemic discrimination privileges cisgender heterosexual survivors.
Hancock et al. (2014), USA	Qualitative (interviews)	Mental health professionals (*n* = 10)	Counseling, art therapy, psychology, social work	IPV assumed to be the same in LGBTQ and heterosexual relationships; lack of intersectionality awareness; inadequate LGBTQ-specific training in counseling programs.
Lim et al. (2024), AUS	Qualitative (interviews)	Participants’ occupational experiences included managerial, advocacy, and clinician roles in refuge accommodation, counseling, primary prevention, advocacy, homelessness support, mental health and alcohol or other drug use services (*n* = 21)	General population service organizations or women-only services, and community-led service organizations	Resistance to inclusive practices; resource constraints; lack of political will and policy support for LGBTQ IPV services.
Simpson and Helfrich (2005), USA	Qualitative (interviews)	Staff members from social service and domestic violence agencies (*n* = 6)	Social service and domestic violence agencies including counseling, crisis intervention, and outreach services)	Systemic heterosexism in policies, training, and service provision; institutional barriers limit IPV support for LGBTQ survivors.

## Study Characteristics

Of the total studies included (*n* = 19), most were conducted in the United States (*n* *=* 10), followed by Australia (*n* = 3), the United Kingdom (*n* = 3), Canada (*n* = 2), and New Zealand (*n* = 1). Most studies were qualitative (*n* = 9), with sample sizes ranging from 5 to 32. The quantitative studies (*n* = 5) had sample sizes between 163 and 1,947, while the mixed-methods studies (*n* = 5) ranged from 256 to 2,482. Various primary care settings were included across the 19 studies. Most studies (*n* = 10) included mental health, counseling, or therapeutic services. Others focused on specialized IPV-related healthcare (*n* = 5), medical services (*n* = 3), or social and community services (*n* = 3). One study broadly referred to primary care services, while others *(n* = 3) described healthcare or support services without specifying exact primary care settings; however, participants in these cases shared experiences or findings relevant to primary care contexts.

## Characteristics of Studies Investigating Barriers to Primary Care Access

Of the studies that investigated barriers to LGBTIQA+ individuals accessing primary care services (*n* = 13), participants’ ages ranged from 16 to 85 (*n* = 9), with the remaining studies (*n* = 4) not reporting participants’ age. Most studies included gay (*n* = 11), lesbian (*n* = 10), or bisexual (*n* = 11) participants. Heterosexual/straight, (*n* = 7) pansexual (*n* = 6), queer (*n* = 6), or asexual (*n* = 5), or Takatāpui (Māori term meaning “intimate companion of the same sex,” used to describe Māori individuals who identify as LGBTQIA+; Kerekere, 2017) participants (*n* = 1) were also included across the studies. Some studies did not report participants’ sexual orientation, or it was unknown or unspecified (*n* = 6).

Most studies included participants who identified as man/male (*n* = 10) or woman/female (*n* = 9), with some specifying cisgender men (*n* = 3) or cisgender women (*n* = 1). Participants who identified as transgender were included in nine studies (*n* = 9), with some specifying transgender men (*n* = 5), transgender women (*n* = 5), assigned male at birth (AMAB) (*n* = 1), or assigned female at birth (AFAB) (*n* = 1). Other studies included participants with unspecified or other gender identities (*n* = 9), with some specifying genderqueer (*n* = 4), agender or gender fluid (*n* = 2), gender nonconforming (*n* = 1), questioning (*n* = 1), Takatāpui (*n* = 1), or bigender (*n* = 1). Nonbinary participants were included in four studies (*n* = 4), with one study specifying nonbinary AMAB and nonbinary AFAB participants.

Various cultural identities and ethnicities were reported. Most studies included participants who identified as Asian (*n* = 10), with one specifying Chinese (*n* = 1), as well as Caucasian, White, or Anglo-Australian (*n* = 9), and those identified as people of color, mixed heritage or ethnicity, multiracial, bi-ethnic, or other (*n* = 9). Participants who identified as Black (*n* = 7) or Hispanic, Latino, Latina, or Latinx (*n* = 7) were also included. Some studies included participants who identified as Native, Native American, American Indian, Native Hawaiian, or Alaskan Native (*n* = 5), African or African American (*n* = 4), or Pacific Islander or Pasifika (*n* = 4), with one specifying Māori (*n* = 1). Participants who identified as Middle Eastern or Arab American were included in two studies (*n* = 2), and Aboriginal and/or Torres Strait Islander participants were included in one study (*n* = 1).

## Characteristics of Studies Investigating Barriers to Providing Support

Of the studies that investigated barriers to providing primary care support for LGBTIQA+ individuals (*n* = 6), only half (*n* *=* 3) reported on participants’ ages, which ranged from 18 to 59. Some studies included gay (*n* = 2), lesbian (*n* = 2), bisexual (*n* = 1), and heterosexual/straight (*n* = 3), with others not reporting on the sexual orientations of the participants (*n* = 3). Across these studies, most included participants who identified as women/female (*n* = 5), with some specifying cisgender women (*n* = 2). Others included participants who identified as man/male (*n* = 3), with one specifying cisgender men (*n* = 1). Some studies did not report on the gender of participants (*n* = 2). A smaller number included participants who identified as nonbinary or transgender, including trans woman, trans man, and transmasculine (*n* = 1), or as a two-spirited woman (*n* = 1). Across these studies, most included participants who identified as Caucasian or White (*n* = 4). Some included participants who identified as Hispanic or Latin (*n* = 2), African American (*n* = 2), Asian, including Southeast Asian, East Asian, or South Asian (*n* = 2), Indigenous or First Nations (*n* = 2), or did not report cultural identities or ethnicities (*n* = 2). A smaller number included participants who identified as Black (*n* = 1), East Indian (*n* = 1), or Middle Eastern (*n* = 1).

## Barriers to Accessing Primary Care Services

Across the 13 studies, barriers to help seeking among LGBTIQA+ individuals, structural influences impacting access to competent primary care support, and exclusionary practices within primary care settings emerged as key barriers to accessing primary care services for IPV support.

## Barriers to Help Seeking

All 13 studies reported that LGBTIQA+ individuals experience significant barriers when seeking help from primary care services for IPV. A common issue identified was the difficulty experienced in recognizing their own experiences as IPV, which then impacted help seeking (D’Cruz et al., 2024; Donne et al., 2018; Donovan & Barnes, 2020; Kurdyla, 2023; Ovenden et al., 2019; Richardson et al., 2015; The Australian Centre for Social Innovation, 2018). Societal narratives that frame IPV within a cisgender and heteronormative context contributed to this misrecognition or minimization of experiences of IPV (Donovan & Barnes, 2020; Kurdyla, 2023; Ovenden et al., 2019; Scheer et al., 2023). Scheer et al. (2023) specifically noted that minimizing IPV was the most common help-seeking barrier among sexual and gender minority youth.

Fear of discrimination and judgment from primary care providers was also a key barrier that impacted help seeking (D’Cruz et al., 2024; Das et al., 2022; Donovan & Barnes, 2020; Kurdyla, 2023; Morris et al., 2024; Palich et al., 2024; Richardson et al., 2015; Scheer et al., 2023). Specifically, the lack of help seeking for IPV support was to avoid and prevent judgment or further marginalization (D’Cruz et al., 2024; Donovan & Barnes, 2020; Turell & Cornell-Swanson, 2005). Some studies also reported that individuals internalized societal stigma around experiencing IPV, leading to feelings of self-blame and inadequacy, which further impacted their willingness to seek help (Donne et al., 2018; Richardson et al., 2015). D’Cruz et al. (2024) and Turell and Cornell-Swanson (2005) also found that LGBTIQA+ individuals avoided disclosing IPV due to concerns about reinforcing negative stereotypes.

Practical barriers, including financial constraints, scheduling challenges, and uncertainty about confidentiality, were frequently reported (Donne et al., 2018; Martin et al., 2022; Palich et al., 2024; Richardson et al., 2015; The Australian Centre for Social Innovation, 2018). For example, Donne et al. (2018) identified that financial costs and insurance gaps significantly inhibited help seeking.

## Systemic Inequities in Primary Care

Across the studies, LGBTIQA+ individuals frequently reported receiving poor and inadequate primary care for their IPV concerns, with systemic inequities identified as key contributors (D’Cruz et al., 2024; Das et al., 2022; Donovan & Barnes, 2020; Martin et al., 2022; Morris et al., 2024; Ovenden et al., 2019; Palich et al., 2024; The Australian Centre for Social Innovation, 2018). A key issue was the absence of inclusive services tailored to the needs of LGBTIQA+ individuals, which often resulted in care that prioritized cisgender and heteronormative contexts (D’Cruz et al., 2024; Donovan & Barnes, 2020; Martin et al., 2022; Morris et al., 2024; The Australian Centre for Social Innovation, 2018). For example, Morris et al. (2024) reported that LGBTIQA+ individuals were screened using a process only suited for cisgender and heterosexual clients, leading to misrecognition of IPV as it uniquely presents in LGBTIQA+ contexts. Further, Das et al. (2022) and The Australian Centre for Social Innovation (2018) reported the absence of LGBTIQA+- suitable referral pathways, which contributed to delays in addressing their IPV concerns.

Many studies also highlighted gaps in provider education and training when supporting LGBTIQA+ individuals who experience IPV, leaving LGBTIQA+ individuals feeling excluded and alienated within primary care settings (D’Cruz et al., 2024; Das et al., 2022; Donovan & Barnes, 2020; Morris et al., 2024; Ovenden et al., 2019; Palich et al., 2024). Specifically, D’Cruz et al. (2024) and Palich et al. (2024) noted that primary care providers lacked knowledge and competency in addressing the unique IPV experiences of LGBTIQA+ individuals, which often resulted in the misidentification of IPV or inappropriate referrals. Further, Ovenden et al. (2019) identified that primary care providers often dismissed IPV as less serious in LGBTIQA+ populations.

## Discrimination, Stigma, and Lack of Cultural Competency

Studies consistently reported that discriminatory and exclusionary practices within primary care settings acted as a significant barrier to LGBTIQA+ individuals seeking IPV support (D’Cruz et al., 2024; Das et al., 2022; Donovan & Barnes, 2020; Kurdyla, 2023; Morris et al., 2024; Palich et al., 2024; Richardson et al., 2015; Scheer et al., 2023; The Australian Centre for Social Innovation, 2018). Anticipated and actual experiences of discrimination, disrespect, and stigma were often described, which created a sense of unsafety and deterred LGBTIQA+ individuals from accessing necessary care (D’Cruz et al., 2024; Das et al., 2022; Donovan & Barnes, 2020; Kurdyla, 2023; Morris et al., 2024; Palich et al., 2024; Richardson et al., 2015; Scheer et al., 2023). Transphobia was specifically reported across two studies, with transgender and gender-diverse individuals experiencing misgendering, denial of gender-affirming care, and dismissal of their identities (Donovan & Barnes, 2020; Kurdyla, 2023). LGBTIQA+ individuals reported feeling judged, blamed, or dismissed by primary care providers (D’Cruz et al., 2024; Das et al., 2022; Donovan & Barnes, 2020; Kurdyla, 2023; Morris et al., 2024; Palich et al., 2024; Richardson et al., 2015; Scheer et al., 2023; The Australian Centre for Social Innovation, 2018). Two studies also reported that LGBTIQA+ individuals concealed their sexual orientation or gender identity to receive support without potential discrimination (D’Cruz et al., 2024; The Australian Centre for Social Innovation, 2018).

## Barriers to Providing Primary Care Services

Across the six studies, several key barriers were identified that impact primary care providers in delivering appropriate support to LGBTIQA+ individuals experiencing IPV. Three key findings emerged from the analysis: (a) heteronormative and cisgendered assumptions, (b) lack of training, knowledge, and resources, and (c) institutional and political resistance to inclusive practices.

## Heteronormative and Cisgendered Assumptions

Studies reported that primary care providers often operated within a framework of heteronormative and gendered assumptions, which limit their ability to recognize and respond effectively to IPV among LGBTIQA+ individuals (Bytheway & Stephens-Lewis, 2024; Furman et al., 2017; Hancock et al., 2014; Simpson & Helfrich, 2005). As a result, IPV as experienced by LGBTIQA+ individuals was overlooked or misunderstood, leading to inadequate screening, misidentification of abuse, and inappropriate interventions (Bytheway & Stephens-Lewis, 2024; Furman et al., 2017; Hancock et al., 2014; Simpson & Helfrich, 2005). These studies also highlighted that providers often lacked awareness of the unique dynamics of IPV within LGBTIQA+ relationships, which contributed to a primary care environment where LGBTIQA+ individuals felt unsupported, reinforcing barriers to disclosure and engagement with services (Bytheway & Stephens-Lewis, 2024; Furman et al., 2017; Hancock et al., 2014; Simpson & Helfrich, 2005). For example, Bytheway and Stephens-Lewis (2024) described how providers placed the burden of educating staff on LGBTIQA+ IPV dynamics onto clients. Furman et al. (2017) also highlighted that primary care providers frequently ignored the influence of intersectionality and societal pressures, which are critical in understanding IPV in LGBTIQA+ relationships.

## Lack of Training, Knowledge, and Resources

Studies consistently reported that primary care providers felt unprepared to support LGBTIQA+ individuals experiencing IPV due to insufficient training and a lack of appropriate resources (Bytheway & Stephens-Lewis, 2024; Du Mont et al., 2024; Furman et al., 2017; Hancock et al., 2014; Simpson & Helfrich, 2005). For example, some providers reported that their formal education did not include specific curriculum content on LGBTIQA+ health needs, resulting in a limited understanding of IPV in these populations (Bytheway & Stephens-Lewis, 2024; Du Mont et al., 2024). Without proper training, providers were often unaware of the distinct experiences of LGBTIQA+ individuals, leading to the misapplication of standard IPV care models that do not account for diverse relationship structures seen in LGBTIQA+ contexts (Bytheway & Stephens-Lewis, 2024; Du Mont et al., 2024; Furman et al., 2017; Hancock et al., 2014; Simpson & Helfrich, 2005).

In addition to educational gaps, studies highlighted the lack of resources tailored to LGBTIQA+ individuals, such as appropriate screening tools and referral pathways (Du Mont et al., 2024; Furman et al., 2017; Simpson & Helfrich, 2005). Specifically, Furman et al. (2017) reported the lack of culturally competent service guidelines, which impacted providers’ ability to create inclusive primary care spaces. Bytheway and Stephens-Lewis (2024) noted that these gaps not only limit professional competency but also discourage disclosure and engagement from LGBTIQA+ individuals. Providers also reported feeling frustrated by the absence of inclusive materials and expressed concerns that this limited their ability to offer meaningful support (Du Mont et al., 2024; Furman et al., 2017; Simpson & Helfrich, 2005).

## Institutional and Political Resistance to Inclusive Practices

Studies identified institutional and political barriers within primary care settings that impacted the implementation of inclusive practices for LGBTIQA+ individuals experiencing IPV (Hancock et al., 2014; Lim et al., 2024; Simpson & Helfrich, 2005). Resistance in organizations to change was often rooted in entrenched institutional norms that prioritize traditional, binary models of care, making it difficult to introduce LGBTIQA+-inclusive policies and practices (Hancock et al., 2014; Lim et al., 2024; Simpson & Helfrich, 2005). Lim et al. (2024) found that while there was sometimes interest to implement LGBTIQA+-inclusive practices, these efforts were routinely constrained by limited time and financial resources. Inclusive service delivery was frequently positioned as supplementary rather than essential, particularly within already overburdened general population services (Lim et al., 2024). Simpson and Helfrich (2005) similarly reported that institutional policies often perpetuated heterosexism. For instance, standard intake and referral forms typically assumed heterosexual partnerships, with “next-of-kin” fields limited to “spouse” or “female partner,” thereby reinforcing exclusionary practices. Furman et al. (2017) further emphasized that inadequate funding and lack of institutional support prevented the implementation of inclusive training programs.

## Discussion

This scoping review identified three key barriers from studies focusing on LGBTIQA+ individuals’ experiences with primary care services for IPV, and three barriers from studies examining the experiences of primary care providers delivering support to LGBTIQA+ individuals experiencing IPV. Table 3 summarizes these findings. Synthesizing and interpreting all six barriers together reveals key insights, and these are discussed below.

**Table 3.** Summary of Critical Findings.

Barriers to Accessing IPV Support for LGBTIQA+ Individuals
- Difficulties in recognizing IPV, or minimizing IPV experiences due to dominant heteronormative narratives, which often led to delayed help seeking. - Fear of discrimination, stigma, and judgment from primary care providers, as well as concerns about reinforcing negative stereotypes, discouraged support seeking
- Systemic inequities, such as the absence of inclusive services, inadequate provider training and education, and a reliance on cisgender, heterosexual frameworks, resulted in inappropriate referrals, delays in support, and culturally unsuitable care
- Discriminatory and exclusionary practices, such as direct and indirect discrimination, led individuals to feel unsafe, judged, and blamed by primary care providers
**Primary care provider barriers to providing support for LGBTIQA+ individuals experiencing IPV**
- Heteronormative and cisgendered assumptions shape primary providers’ approaches to IPV support, often leading to misidentification, inadequate screening, and ineffective interventions
- A lack of education, training, and resources left many providers unprepared to address the unique needs of LGBTIQA+ individuals’ experiences of IPV, leading to the use of generic IPV support approaches ill-suited for LGBTIQA+ individuals
- Institutional and systemic resistance, driven by policy constraints, limited funding, and competing priorities, was reported to impact the implementation of suitable primary care support for LGBTIQA+ individuals experiencing IPV

Our findings indicate that overarching heteronormative and cisgendered assumptions related to IPV not only prevent LGBTIQA+ individuals from recognizing and understanding their IPV experiences (Das et al., 2022; Donne et al., 2018; Donovan & Barnes, 2020; Kurdyla, 2023; Ovenden et al., 2019; Richardson et al., 2015; The Australian Centre for Social Innovation, 2018) but also limit primary care providers’ ability to identify and respond appropriately (Bytheway & Stephens-Lewis, 2024; Furman et al., 2017; Hancock et al., 2014; Simpson & Helfrich, 2005). The pervasive presence of these exclusionary assumptions within healthcare and primary care systems has been well established in the literature (Bermea et al., 2018; Bytheway & Stephens-Lewis, 2024; Calton et al., 2016; Donne et al., 2018; Lim et al., 2024; Wright et al., 2022). Specifically, in their review, Calton et al. (2016) identified that dominant cisgendered and heteronormative structures significantly contribute to the misrecognition and minimization of IPV within LGBTIQA+ relationships, reinforcing barriers to help seeking for IPV and the quality-of-service provision offered. The current review echoes existing literature in calling for the urgent dismantling of cisgendered and heteronormative structures within primary care systems. Specifically, it highlights the need for such reforms within primary care services to improve both the recognition of IPV among LGBTIQA+ individuals and the capacity of primary care providers to respond effectively.

Systemic inequities, such as inadequate training, a lack of inclusive resources, and institutional resistance, were evident, further compounding challenges for both LGBTIQA+ individuals and primary care providers (D’Cruz et al., 2024; Das et al., 2022; Donovan & Barnes, 2020; Martin et al., 2022; Morris et al., 2024; Ovenden et al., 2019; Palich et al., 2024; The Australian Centre for Social Innovation, 2018). For LGBTIQA+ individuals, these barriers manifest as delayed help seeking due to factors such as fear of discrimination and internalized stigma (D’Cruz et al., 2024; Das et al., 2022; Donovan & Barnes, 2020; Martin et al., 2022; Morris et al., 2024; Ovenden et al., 2019; Palich et al., 2024; The Australian Centre for Social Innovation, 2018). For primary care providers, they contribute to a reliance on outdated care models and a lack of culturally informed protocols (Bytheway & Stephens-Lewis, 2024; Du Mont et al., 2024; Furman et al., 2017; Hancock et al., 2014; Simpson & Helfrich, 2005). These findings are reinforced in a review by Santoniccolo et al. (2023), who reported that insufficient provider training and a lack of tailored services deter LGBTIQA+ individuals from seeking IPV support. Similarly, a review by Freedberg (2006) found that systemic barriers, including limited LGBTIQA+ competency training, exacerbate the reluctance of LGBTIQA+ individuals to engage with services due to concerns about stigma and inadequate care. These parallels with the current review highlight the urgent need for systemic reform, comprehensive LGBTIQA+ training, and inclusive resources such as tailored screening tools to create an inclusive and supportive primary care service for LGBTIQA+ individuals experiencing IPV.

The reciprocal nature of the identified barriers for both LGBTIQA+ individuals and primary care providers necessitates a dual-targeted approach to improving IPV primary care service provision. Strategies are needed to encourage and empower LGBTIQA+ individuals to recognize their experiences as IPV and to seek support accordingly (D’Cruz et al., 2024; Das et al., 2022; Donovan & Barnes, 2020; Kurdyla, 2023; Morris et al., 2024; Palich et al., 2024; Richardson et al., 2015; Scheer et al., 2023). In addition, primary care providers should have access to and undergo ongoing comprehensive training and systemic reforms that support them to offer inclusive, culturally competent IPV primary care (Hancock et al., 2014; Lim et al., 2024; Simpson & Helfrich, 2005). Previous literature supports this dual approach, with Wright et al. (2022) emphasizing that effective IPV interventions must address both individual-specific barriers and provider preparedness, while Calton et al. (2016) argue that without targeted efforts to enhance LGBTIQA+ individuals’ access to care and equip providers with the necessary skills and knowledge, IPV support services will remain inadequate.

## Assessment of Diversity and Inclusion

The studies included in this review represent a diverse range of LGBTIQA+ identities, including gay, lesbian, bisexual, transgender, nonbinary, queer, and Takatāpui individuals (D’Cruz et al., 2024; Das et al., 2022; Donne et al., 2018; Donovan & Barnes, 2020; Kurdyla, 2023; Martin et al., 2022; Morris et al., 2024; Ovenden et al., 2019; Palich et al., 2024; Richardson et al., 2015; Scheer et al., 2023; The Australian Centre for Social Innovation, 2018; Turell & Cornell-Swanson, 2005). It is also acknowledged that synthesizing findings across studies can involve aggregating LGBTIQA+ populations, which can limit the ability to fully consider and report on the unique barriers and experiences of specific subgroups. Furthermore, none of the studies involved people who are intersex. This omission is concerning given the unique service needs and IPV experiences that intersex individuals may face, including medical coercion, lack of bodily autonomy, and systemic exclusion from LGBTIQA+ service frameworks (Campo & Tayton, 2015; Zeeman & Aranda, 2020). Future research should directly recruit and involve intersex individuals to ensure that specific primary care barriers to IPV support are identified and addressed.

The studies captured a wide range of racial and ethnic diversity, including Caucasian, Black, African or African American, Hispanic, Latino, Latina, or Latinx, Asian, Pacific Islander or Pasifika, Māori, Native American, Aboriginal, and/or Torres Strait Islander, and Middle Eastern participants. However, across the studies, there was limited and inconsistent disaggregation of data to examine how intersecting racial and cultural factors influenced IPV experiences and access to primary care. This gap highlights the need for research that directly addresses the unique barriers faced by LGBTIQA+ individuals at the intersection of IPV and race and ethnicity.

Geographically, the majority of the 19 studies were conducted in the United States, with fewer conducted in Australia, the United Kingdom, Canada, and New Zealand. The dominance of U.S.-based studies limits the applicability of the findings to other countries, where healthcare systems, legal protections, and IPV services may differ. For example, in Australia, research on LGBTIQA+ experiences of IPV within primary care remains limited. In addition, Australia’s vast geographic diversity means that LGBTIQA+ individuals in rural and remote areas often encounter distinct challenges, including limited access to specialized services, a greater risk of stigma in smaller communities, and fewer LGBTIQA+ inclusive healthcare providers (Amos et al., 2023; Hill et al., 2020, 2021).

## Implications for Research, Practice, and Policy

The implications for research, practice, and policy that emerged from this review are discussed below and summarized in Table 4.

**Table 4.** Summary of Implications for Research, Practice, and Policy.

Type	Implications
Research	Prioritize co-design of inclusive interventions with LGBTIQA+ individuals experiencing IPV Critically assess and audit primary care services for systemic inequities. Implement research-based, culturally appropriate solutions for accessible IPV primary care support.
Practice	Transformative change to the IPV support provided for LGBTQIQA+ individuals needed in primary care services. Mandatory LGBTIQA+ inclusivity training to challenge cisnormativity and build culturally safe models of care. Extend screening tools and referral pathways, co-designed with LGBTIQA+ individuals.
Policy	Mandate LGBTIQA+ culturally competent care guidelines with accountability measures. Targeted funding for IPV support in LGBTIQA+ populations is essential for training and evaluation. Foster collaborative partnerships between primary care providers, policymakers, and LGBTIQA+ organizations.

The findings of this review, alongside other similar reviews (Calton et al., 2016; Freedberg, 2006; Santoniccolo et al., 2023; Wright et al., 2022), confirm the existence of significant barriers to primary care access and support for LGBTIQA+ individuals experiencing IPV, leading to important research implications. Given the significant need for primary care services to be better equipped to support LGBTIQA+ individuals experiencing IPV, future research should prioritize the formal development, co-design, and evaluation of inclusive interventions and strategies. It is essential that LGBTIQA+ individuals have a voice in this process, ensuring that primary care services are responsive to their specific needs. In addition, given the pervasive influence of heteronormative and cisgendered assumptions in primary care systems, research must critically assess and audit primary care services to identify systemic inequities that contribute to the exclusion of LGBTIQA+ individuals. Addressing these gaps requires the implementation of research and evidence-based culturally appropriate solutions that build more LGBTIQA+ inclusive and accessible IPV primary care support.

Similarly, practice implications that emerged from this review’s findings concern the need for transformative change in primary care services. This includes the mandatory ongoing, comprehensive LGBTIQA+ inclusivity training that challenges cisgendered and heteronormative assumptions and builds skills in culturally safe primary care. Furthermore, updating screening tools and referral pathways, ideally co-designed with LGBTIQA+ individuals, will tailor primary care to the unique needs of these populations (Ovenden et al., 2019; The Australian Centre for Social Innovation, 2018).

The policy implications arising from this review include the need for mandating the adoption of LGBTIQA+ culturally competent care guidelines within primary care services, along with accountability measures to ensure these guidelines are adhered to (Lim et al., 2024). Additionally, targeted funding focused on increasing the responsiveness of primary care services to IPV within LGBTIQA+ populations is crucial for supporting training, resource development, and the ongoing evaluation of interventions (Calton et al., 2016; Wright et al., 2022). It is also essential for fostering collaborative partnerships between healthcare providers, policymakers, and LGBTIQA+ community organizations (Calton et al., 2016; Wright et al., 2022).

## Strengths, Limitations, and Future Directions

This scoping review is the first to specifically examine the barriers faced by both LGBTIQA+ individuals in accessing primary care support and primary care providers in delivering IPV support. As such, the key insights and implications are focused on the primary care context, which is a novel contribution to existing literature. In addition, synthesizing findings from both LGBTIQA+ individuals and primary care providers offers a holistic view of the systemic challenges that impact effective primary care responses. A rigorous search strategy was employed, utilizing multiple academic databases and gray literature sources, while adherence to the JBI methodology ensured systematic data extraction and thematic synthesis.

While this review focused on barriers for primary care providers, it did not include all types of primary care providers. Most studies focused on mental health professionals, therapists, and social or community service providers, with limited representation of general practitioners, nurses, and other medical professionals who are often the first point of contact for individuals experiencing IPV. The absence of these perspectives limits our understanding of how primary care operates as the initial point of engagement for LGBTIQA+ individuals experiencing IPV. Future research should explore the experiences of these primary care providers to gain a more comprehensive view. Regarding the studies that examined primary care providers offering IPV support, only one was quantitative. To capture a broader range of experiences, future research should incorporate more quantitative methods. In addition, there was a notable lack of studies exploring primary care providers’ experiences delivering support for IPV. Many studies did not report on the specific roles and service settings of providers, making it unclear whether certain studies were relevant to this review. To ensure adherence to the inclusion criteria, these studies had to be excluded. It is acknowledged that some important studies may have been missed as a result. Future research should clearly report the professional roles of providers and the settings in which they work, as this will be essential to capturing the full scope of barriers in delivering IPV primary care support. Furthermore, the lack of non-U.S. studies highlights the need for more research to better understand how IPV is experienced and addressed globally.

## Conclusion

This scoping review highlights the complex interplay of barriers that impact both LGBTIQA+ individuals’ access to, and primary care providers’ ability to deliver, effective IPV support. Overarching cisgender and heteronormative assumptions, alongside systemic inequities, create a reciprocal cycle of challenges. This calls for targeted, dual-focused interventions that not only encourage LGBTIQA+ individuals to seek primary care support but also ensure that this support is culturally appropriate. By integrating insights from both LGBTIQA+ individuals and primary care providers, this review highlights the urgent need for primary care services that are both inclusive and culturally responsive, ultimately improving outcomes for all LGBTIQA+ individuals experiencing IPV.

## Supplemental Material

sj-docx-1-tva-10.1177_15248380251361063 – Supplemental material for Barriers to Accessing and Providing Primary Care Services for LGBTIQA+ Individuals Experiencing Intimate Partner Violence: A Scoping Review Supplemental material, sj-docx-1-tva-10.1177_15248380251361063 for Barriers to Accessing and Providing Primary Care Services for LGBTIQA+ Individuals Experiencing Intimate Partner Violence: A Scoping Review by Jack Farrugia, Danny Della Vedova, Bronwyn Milkins, Jacqueline Hendriks, Sharyn Burns and Roanna Lobo in Trauma, Violence, & Abuse
