# Supplementary material for: Barriers to Accessing and Providing Primary Care Services for LGBTIQA+ Individuals Experiencing Intimate Partner Violence: A Scoping Review
Source: Trauma Violence Abuse. 2025 Aug 30;27(4):1848–62. doi: 10.1177/15248380251361063 (PMC13578645; doi:10.1177/15248380251361063)
Supplement: Supplementary file 1 [file sj-docx-1-tva-10.1177_15248380251361063.docx]

Appendix A

ProQuest Search Strategy

(((noft("reproduct* coercion" OR "reproduct* abuse" OR RCA OR "coerced reproduction" OR "reproductive control" OR "pregnancy coercion" OR IPV OR "spous* abuse" OR "domestic* control*" OR IPV) AND PEER(yes)) OR (noft(violen* NEAR/3 (intimate OR partner* OR domestic OR family OR dating OR gender*)) AND PEER(yes)) OR (su("Intimate partner violence" OR "Domestic violence" OR "Partner abuse" OR "sexual abuse") AND PEER(yes))) AND ((noft(lesbian* OR gay OR homosexual* OR bisexual* OR queer OR intersex OR asexual* OR pansexual* OR agender OR demisexual OR graysexual OR transgender* OR ("trans gender") OR transsexual* OR transpeople OR "trans* people" OR "trans* person*" OR "trans* men" OR "trans* man" OR transguy OR "trans* women" OR "trans* woman" OR transgirl OR "gender diverse" OR "gender queer" OR agender OR bigender OR "bi gender" OR genderfluid OR "gender fluid" OR transmasculine OR "trans masculine" OR "gender non-conforming") AND PEER(yes)) OR (su("LGBTQ people" OR "Gender identity") AND PEER(yes)))) AND stype.exact("Conference Papers & Proceedings" OR "Scholarly Journals" OR "Dissertations & Theses") AND (stype.exact("Scholarly Journals") AND PEER(yes))

Search was limited to 2004-2024, English language only, and published in an OECD country.
